# Prediction of Cow Calving in Extensive Livestock Using a New Neck-Mounted Sensorized Wearable Device: A Pilot Study

**DOI:** 10.3390/s21238060

**Published:** 2021-12-02

**Authors:** Carlos González-Sánchez, Guillermo Sánchez-Brizuela, Ana Cisnal, Juan-Carlos Fraile, Javier Pérez-Turiel, Eusebio de la Fuente-López

**Affiliations:** ITAP (Instituto de las Tecnologías Avanzadas de la Producción), Universidad de Valladolid, Paseo del Cauce 59, 47011 Valladolid, Spain; cgonzalezs90@gmail.com (C.G.-S.); guillermo.sanchez.brizuela@uva.es (G.S.-B.); ana.cisnal@uva.es (A.C.); turiel@eii.uva.es (J.P.-T.); efuente@eii.uva.es (E.d.l.F.-L.)

**Keywords:** cow, extensive livestock, sensorized wearable device, monitoring, parturition prediction

## Abstract

In this study, new low-cost neck-mounted sensorized wearable device is presented to help farmers detect the onset of calving in extensive livestock farming by continuously monitoring cow data. The device incorporates three sensors: an inertial measurement unit (IMU), a global navigation satellite system (GNSS) receiver, and a thermometer. The hypothesis of this study was that onset calving is detectable through the analyses of the number of transitions between lying and standing of the animal (lying bouts). A new algorithm was developed to detect calving, analysing the frequency and duration of lying and standing postures. An important novelty is that the proposed algorithm has been designed with the aim of being executed in the embedded microcontroller housed in the cow’s collar and, therefore, it requires minimal computational resources while allowing for real time data processing. In this preliminary study, six cows were monitored during different stages of gestation (before, during, and after calving), both with the sensorized wearable device and by human observers. It was carried out on an extensive livestock farm in Salamanca (Spain), during the period from August 2020 to July 2021. The preliminary results obtained indicate that lying-standing animal states and transitions may be useful to predict calving. Further research, with data obtained in future calving of cows, is required to refine the algorithm.

## 1. Introduction

The study and monitoring of livestock has always been a subject of great interest. Indeed, quantitative measurement of animal behaviour is an important tool for understanding their reproduction, survival, welfare, and interaction with other animals [1]. Animal activity is one of the most important indicators associated with animal health and welfare [2], and animal behaviour is an indicator of the well-being and health of cows [3]. Detecting changes in the behaviour and activity of cows is a good preventive tool to determine the animal’s health status [4].

Every year an average of 8.5% of perinatal calves are lost due to natural abortions, stillbirths, and complications during parturition (calving) [5], which translates into higher economical costs and reduced animal wellbeing. Ideally the calving process should be carefully overviewed by experts to avoid or correct any problems that may arise (e.g., dystocia). Even today, the analysis of cow behaviour and calving detection is mainly carried out by experienced workers through unaided monitoring. These approaches are however expensive and time consuming, and not effective for extensive livestock farming, where many animals are kept under grazing in the open air on large areas of surface.

An automated solution based on cow data collection from sensors could provide better calving predictions. This will allow the farmer to better identify those cows that require intensive supervision and to focus on caring for cows with upcoming calving, reducing possible risks and improving the health and wellbeing of the animals.

Internet of things (IoT), an already mature and effective technology, can help improve the efficiency and productivity in agriculture and livestock production systems [6]. IoT has initially spread into the agriculture and farming industry, and mainly aims to supervise the well-being of animals, thus enhancing the profitability of farms by increasing productivity [7]. Most connected livestock solutions are developed for cattle, especially cows, due to the valuable price of such animals [8].

Precision livestock farming (PLF) aims to manage individual animals by continuously monitoring their health, welfare, production/reproduction and/or environmental impact in real time [9]. This is achieved through real-time image [10,11] and/or sound analyses or by wearable devices with sensors that monitor physical (position, direction of movement, speed…) [12,13,14,15], and physiological variables (heart rate, breathing rate, temperature…) [16,17,18] of each animal.

Different physiological and behavioural parameters associated with calving can be monitored through sensors. The analysis of the internal temperature and its evolution, usually measured in the vulva, the rectum, or the rumen of the animal, is one of the most accurate calving predictors [19]. It has been demonstrated that a decrease in vaginal temperature equal or greater than 0.3 °C in cows bearing singletons can predict calving within the next 36 h in 83.3% of cases and up to 100% within 60 h [20]). However, the core temperature of the animal is difficult to be measured in a non-intrusive way, and the available commercial solutions require intensive veterinary care to install and to check the correct location of the measuring device. This approach is therefore not preferred for use in PLF.

An exhaustive meta-analysis of the different publications related to calving detection in cows is showed in [21]. It concludes that automated monitoring and detection of calving, as well as of dystocia incidents, is possible. However, behavioural changes associated with calving vary between individual animals. Behaviour associated with feeding and rumination descent gradually in the two weeks leading up to calving and is drastically reduced during calving [22]. The duration of rumination descends up to 33% the day when calving takes place in comparison with the previous day. This behaviour could be successfully measured using ear or neck-mounted devices [23,24,25,26].

Another indicator of calving is the increase in lying bouts (LB). This behaviour is associated with the restlessness that the animal feels due to the imminency of the calving. The frequency of lying bouts and their mean duration increase greatly as the calving event approaches, starting already 48 h before and being maximized on the day of calving [26]. This increase [6] (from 9.3 ± 1.31 LB/day four days before calving to 13.0 ± 1.02 LB/day the day of calving) is especially important in heifers, but also multiparous dam show more activity prior to calving, and can be observed on average 6 h before calf birth [27].

Lying bouts can be easily identified using a leg-mounted accelerometer [28], but detection is much more challenging using ear or neck-based sensors due to the similarities in the signals from the accelerometer when the animal is standing and lying. A recent study [29] reported the use of a neck-based accelerometer to distinguish between those states by detecting the characteristic movement associated with the transition between states. After calving, the number of steps per hour stays elevated, whereas lying bouts tend to gradually decrease as the animal transitions between pre and postpartum states.

Tail-raising patterns have been observed to change in the 24 h prior to calving [30,31] and can be monitored using tail-mounted accelerometers. This new approach is however not viable for long-term monitoring due to the weight limitation of these devices, overall reduced stability, and possible damage to the skin of the animal [32].

In calving prediction by traditional methods, the farmer makes a visual inspection of the cow to know its status. This is an error-prone task in which even experts may fail to provide an accurate prediction of calving date. In extensive farming, animals move freely in a wide area, which makes it more difficult for the farmer to properly monitor and manage pregnant cows. An automated solution based on cow data collected from sensors and processed by algorithms, can provide better delivery predictions than visual observation. This will allow the farmer to have a more accurate estimation of the expected calving date of the cow and to identify those cows that require intensive supervision due to the proximity of calving. It will make possible both reducing the workload of farmers, who can focus on caring for cows with upcoming calving and improving the health of the cow.

In this paper a low-cost neck-mounted sensorized wearable device was designed and an algorithm was developed to detect the onset of calving of cows in extensive livestock farming. This work was divided in three main phases: (1) development of a wearable solution for data collection based on different sensors; (2) data collection in extensive livestock farming by using the aforementioned solution and human-based observations; and (3) development of algorithms to detect the onset of calving and creation of a decision function based on the frequency and duration of lying and standing behaviour (lying bouts).

This paper is organized as follows. Section 2 presents the sensorized wearable device, the software developed to collect data from different cows by human observers, gives an overview of the collected data, as well as the methodology followed during this study and introduces the proposed algorithm for parturition detection. Results and discussion of this algorithm are presented in Section 3. Finally, Section 4 shows the conclusions and future work.

## 2. Materials and Methods

The goal of the first phase of the study is the development of a solution for recording large quantities of behavioural information, which will later be combined with human observation of the animals. The captured information will be used for the development, validation, and quantification of algorithms for calving prediction.

A new low-cost sensorized wearable device was developed and integrated into a collar, which can be placed around the cow’s neck. The developed device incorporates three sensors: an Inertial Measurement Unit (IMU), a GNSS and a thermometer. Human observers helped with monitoring, labelling, and recording the animals’ state using our own development PC software. The designed collars were tested on cows from an extensive livestock farm in Salamanca (Spain), during the period August 2020–July 2021.

### 2.1. Sensorised Wearable Device

**Figure 1:**
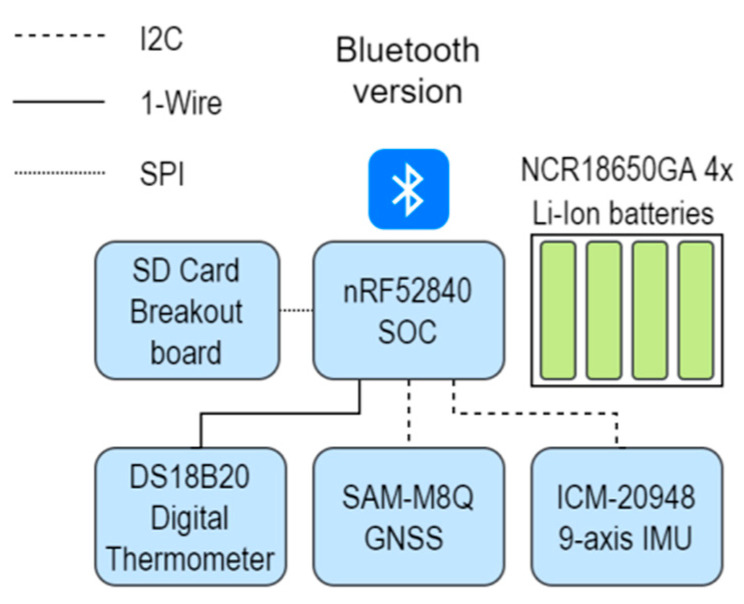
Overall architecture of sensorized collar.

The sensorized wearable device is a collar which is placed on the animal’s neck. The collar houses a nRF52840-dongle (Nordic Semiconductor, Trondheim, Norway) microcontroller, three sensors (thermometer, 9-axis IMU—3-axis accelerometer, 3-axis gyroscope and 3-axis magnetometer—and GNSS), a microSD card breakout board (AdaFruit, NY, EEUU) for data storage and lithium batteries. Figure 1 shows the overall architecture of the collar.

The microcontroller nRF52840 is a small, low-cost device built around the 32-bit ARM^®^ Cortex™-M4 CPU which supports short-range wireless standards including BLE. For measuring the cow’s body temperature, a DS18B20 digital thermometer with a temperature range of −55 to 125 °C (accuracy ± 0.5 °C) is placed in such a way that contact between the skin of the cow and the sensor occurs. The microcontroller acquires the 9–12 bits configurable Celsius temperature measurements using a unique 1-wire interface, which only requires one port pin for the communication. The IMU integrated in the collar is the ICM-20948 (InvenSense, Berkeley, CA, USA, EEUU), which is a low-power 9-axis motion tracking device embedding a 3-axis gyroscope, a 3-axis accelerometer, and a 3-axis compass. A SAM-M8Q (U-blox, Thalwil, Switzerland) receiver is used for precise geographic positioning of the animal and is configured to work in PSM (Power Save Management) mode to minimize power consumption. Both the IMU and the GNSS communicate with the nRF52840 microcontroller using I^2^C at 400 KHz. The microSD card, which allows the storage of data from the sensors, communicates with the microcontroller using SPI. Four lithium ion NCR18650GA (Sanyo, Osaka, Japan) batteries of 3.7 V and 3350 mA were used to power the device.

**Figure 2:**
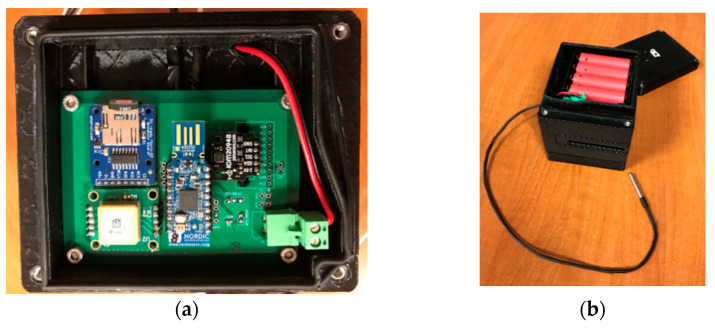

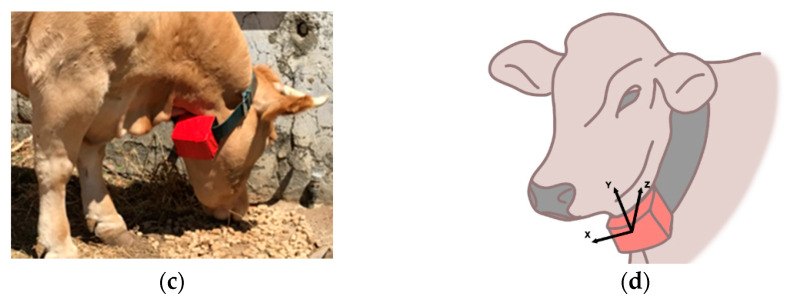
Collar developed for data collection. (**a**) PCB with electronic components; (**b**) collar batteries and temperature sensor; (**c**) collar with belt placed on the cow’s neck; (**d**) IMU axis orientation in the collar.

Figure 2 shows the developed collar. To avoid damage to the electronic components and the batteries, a protective box was designed and produced using additive manufacturing in a 3D printer (Ultimaker^®^ 2+). The polymer selected was ABS, a low-cost plastic material with good mechanical properties. The cover box has two areas (Figure 2a,b). The lower part contains the electronics, whereas the upper area houses the batteries. This design facilitates the replacement of the batteries in the collar without affecting the electronic components. To waterproof the container, a 2 mm diameter nitrile rubber O-ring was fitted in the junction between the two covers. The box was attached to the neck of the cows using an adjustable leather belt (Figure 2c), which allows for a good fit on animals of different sizes. The material of the belt is soft, which maximises the animal’s comfort.

All electronic components of the collar were configured to work in low power mode, to minimize power consumption, and to extend battery life. The data from the accelerometer and the gyroscope embedded in the IMU were sampled at a frequency of 17.6 Hz. The temperature and geolocation information (longitude, latitude, altitude, and speed) were collected at 1 Hz. The orientation of the IMU axis is shown in Figure 2d.

Initial tests were performed using BLE 4.2 for the communication between the board and a computer where data was registered. Due to high power consumption, however, it was decided to store the information onboard instead, using a microSD card. This approach increased the average collar battery life to up to 15 days.

Each datapoint includes the information from the sensors and a timestamp, using the format described in Table 1. The data are then saved to the microSD card using binary format. To minimize data loss if the collar runs out of batteries or fails, a new file is created every hour.

Although the data transmission is no longer performed wirelessly, the collar still makes use of BLE: during collar initialization a timestamp will be exchanged between the collar and the laptop, which allows for later data synchronization. A keepalive message is also sent periodically (every minute), from collar to laptop, using BLE, to allow the human observer monitoring the cow to detect if the collar is still operative or another action is required (e.g., replacement of batteries).

### 2.2. Data Annotation with Computer Software

**Figure 3:**
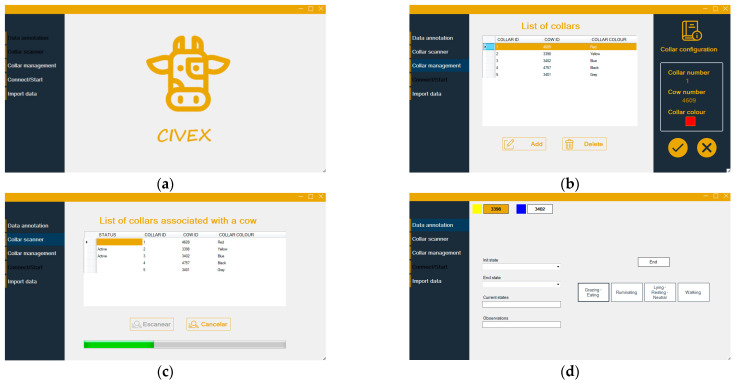
PC Software for collar management and data collection based on visual observation. (**a**) Startup screen; (**b**) collar management; (**c**) collar scanner; (**d**) data annotation.

Direct visual observations were used to collect states and actions of cows in their natural environment at the cattle farm. A PC-based software (Figure 3a) was designed and developed to help the user register the observed state of multiple cows. The program includes the following functionalities:

Collar initialization

During collar initialization, a timestamp is sent to the collar using BLE. This timestamp will be used for the synchronization of the sensor data collected by the collar and the states and actions of the cows recorded by the human observer.

Collar management (Figure 3b)

According to the European Commission, individual identification and registration of bovine livestock is mandatory to ensure full traceability and, consequently, enhance food safety and better safeguard animal health. The application uses an alpha-numeric code as the cowl’s ID. Similarly, each device is identified by a unique 64-bit collar ID, which corresponds to the serial number of the microcontroller housed in the collar.

During operation, it was noted that human observers sometimes had difficulty identifying cows when recording actions, especially over long distances or when animals were close to each other. To solve this, each manufactured collar was printed with a different colour (Figure 2c shows a red collar). Collar management allows registering and assigning a new collar to an animal, unregistering, or reassigning an existing one and updating the colour of the collar.

Collar scanner (Figure 3c)

This feature allows to check active collars within the BLE’s range, by listening to a keepalive message containing the ID that the devices send every minute. The discovered collars are listed on an overview and their status is changed to active (Figure 3c). The application prevents the annotation of data from not active collars.

Data annotation (Figure 3d)

It allows the annotation, by a human observer, of the status and the action the cow is performing. Additional observations can also be recorded. All annotated data and observations are stored on the PC. Once the annotation activity is finished, the software automatically generates a file containing the recorded information. Two predetermined sets of states have been considered:

- General behaviour. The tags considered are: “Grazing/Eating”, “Ruminating”, “Neutral” and “Walking”.
- Standing behaviour. The tags considered are: “Standing” and “Lying” position.

This reduction was necessary to identify transitions between lying and standing positions (lying bouts) which would not be registered in the general behaviour tag set, where the neutral state can happen both standing and lying (if the cow is doing nothing else).

Import data:

Once the operator recovers the collar and obtains the sensor data files stored in the microSD, the application allows the generation of a final file, using the timestamp in both files to combine and synchronize the data from the collar with the data from the file containing the visual observation data (stored in the PC). This synchronized data set is used to develop our own parturition prediction algorithm described in Section 2.3.2.

### 2.3. Calculation

#### 2.3.1. Animals, Facility and Data Collection

For this study, ten collars were developed. However, cow monitoring was limited to a maximum of three animals simultaneously in order to improve the quality of the annotated data. The rest of the produced collars were saved as replacements, to minimize dead times and data loss in case a collar stopped working. The monitored beef cattle belong to an extensive livestock farm located in the municipality of Carrascal de Barregas, in the province of Salamanca, Spain.

During the study, collars were fitted to six cows (see Table 2). For eleven months (August 2020–July 2021) two experienced observers (one working in the morning shift and the other during the afternoon shift) annotated the actions of the animals for a total of 6 h every day. It is to be noted that although the observers were following the animals continuously, some situations introduced uncertainty in the data, for example, when cows stampede from one location to another. To mitigate the data deterioration, observers left the annotations blank when detecting these situations. To record the different states a laptop running the application previously presented was used while maintaining a clear line of sight with the animals. Every week, the data stored in the microSD card of the collar was downloaded, and the batteries were replaced. A detailed overview of the cow-wise distribution of the data is presented in Table 2. As datapoints were dumped in the SD card on an hourly basis, it is straightforward to know the number of hours we got data from. The total quantities shown in Table 2 accumulate the number of hours the collar was recording data (raw data) and the number of hours the observer labelled behaviours for every studied cow.

The schema of data acquired with the collars is shown in Table 3. In total, more than 855 million (855,319,572) raw datapoints have been recorded, of which approximately 114 million (114,167,178) are labelled.

Previous research work related to monitoring of pregnant cows around calving, (Jensen, 2012) and (Titler et al., 2015), has been focused on the period immediately around the time of calving (one and four days, respectively). This approach however is not practical for application where monitoring is less frequent, such as extensive livestock farms where large herds are held. Therefore, our research was focused on the long-term monitoring of the pregnant animals, which extended up to two months after calving. Using this approach, we could analyse the individual behavioural change during different stages of pregnancy, which previous research has proved can differ greatly between individuals [21].

The labels used for data annotation are presented in Table 4 and Table 5. These two sets of labels distinguish the two datasets introduced before, namely general behaviour and standing/lying behaviour. Considering both label sets, approximately 1.777 h of cow behaviour have been annotated by two experienced observers (working part-time in morning and afternoon shifts).

**Figure 4:**
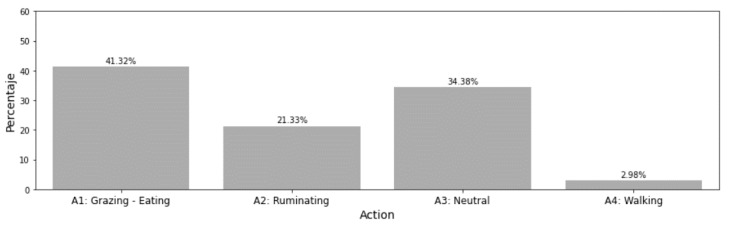
Distribution of general behaviour annotations.

**Figure 5:**
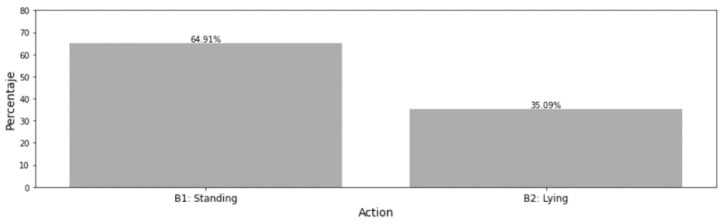
Distribution of standing/lying behaviour annotations.

The distribution of the annotated actions is presented in Figure 4 for general behaviour actions and in Figure 5 for standing/lying behaviour.

General behaviour (Table 4) action prediction [33] has been explored during the study as additional input for calving prediction. Furthermore, data already gathered allows for further investigation in this field without the need of additional human-labelling. However, the general behaviour label set can lead to an imperfect classification of standing/lying behaviour (lying bouts) of the cow when transformed, as some actions can only be carried out in one posture (e.g., walking-standing), but other may occur both standing and lying.

For this reason, a second set of labels (Table 5) only accounting for lying and standing postures has been used to ensure a correct classification of these two postures when needed due to the importance of lying bouts detection for calving detection (as discussed in the introduction).

The annotated data from human observations show a small delay between the change of action of the cow and the annotation of this new action due to the response time of the observer and their need to operate the annotation software. For this reason, the datapoints recorded two minutes before an action change were ignored. This time period was chosen as a balance between loss of data and minimizing mislabelled data in the dataset. As cows did not change actions with high frequency in the recorded labels, two minutes resulted in enough certainty without losing a significative volume of data for each action.

As discussed previously, the sensor readings from the collars and the annotations from the observers can be joined using the timestamp of each data point to form the final dataset.

Three datasets were generated using the recorded data:

- Non-annotated data from the devices. These data have been proved to be useful for unsupervised and semi supervised learning tasks.
- General behaviour annotated data (Table 4). This dataset could be used to classify and predict the animal actions based on new reading from the devices.
- Standing/lying behaviour annotated data (Table 5). This dataset, although smaller compared to b, serves for statistical learning tasks, as well as semi-supervised learning techniques.

The experiments and algorithms have been developed using Python 3.7 [34] along several libraries, mainly: Pandas [35], Keras [36], Numpy [37], and SciPy [38]. Figures have been plotted using the Seaborn [39] library.

#### 2.3.2. Parturition Prediction Algorithm

Calving prediction is the main objective of this pilot study. To usefully notify parturition, it is necessary to detect it with enough anticipation using a low-memory algorithm suited for the microcontroller.

The number of transitions of the animal between lying and standing has been empirically proved to be a good indicator of parturition in different studies [27,40,41]. This measure serves as an indicator of the proximity of calving due to the relative increase of its value in the 8 to 2 h before the parturition event. Furthermore, a notable decrease in the number of lying bouts in the hours after calving is also observed. However, these works study intensive dairy cattle, while our work studies extensive beef cattle, with the according significantly less restricted environment since calving barns are not used. A new low-memory algorithm based on classification of two cow postures (lying and standing), from the collar sensors readings has been developed. To classify these behaviours, accelerometer readings, commonly used to distinguish between lying and standing, as well as GPS altitude readings were initially considered. However, GPS readings were discarded due to insufficient sensor resolution.

**Figure 6:**
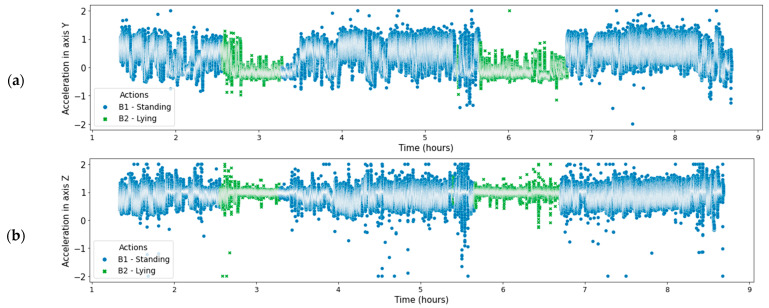
Y-axis and Z-axis accelerometer signal with human-labelled standing and lying annotations. (**a**) Y-axis; (**b**) Z-axis.

In [21] is indicated that is more difficult to distinguish between a lying and standing position with a neck-based accelerometer since the two positions show similar accelerometer readings. Leg-based accelerometers show a distinct crossover of two axes and can easily be utilized to determine a standing or lying position. However, we have analyzed accelerometer signals read by our neck-mounted collars on the Y and Z axes (Figure 6). This figure shows that the analysis of the accelerometer signals provided by the cow’s collar, allow us to clearly distinguish cow lying position (green colour), and cow standing position (blue colour). These results are similar to those presented in [29] to classify the cow posture as standing or lying.

**Figure 7:**
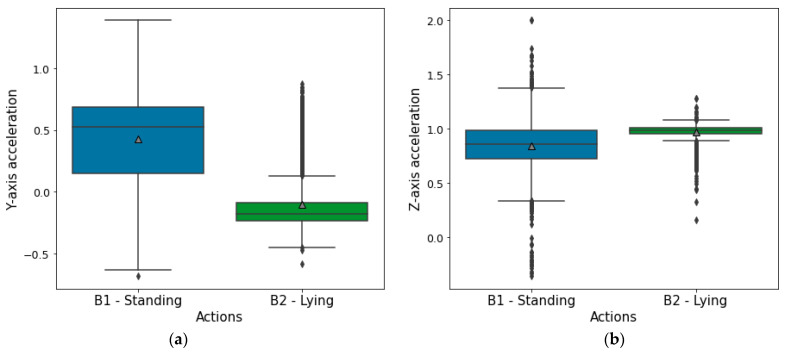
Y-axis and Z-axis accelerometer signal distribution while standing and lying of all data annotated with standing and lying postures. (**a**) Y-axis; (**b**) Z-axis.

Our algorithm for cow posture classification (lying/standing) based on accelerometer readings has been implemented based on an heuristic threshold [27]. This approach is focused on simplicity and is based on the different distributions of acceleration recordings along the Y axis depending on the posture of the animal. Figure 7 shows the distribution of all available data labelled with the standing/lying action (11 months of data collection). Accelerometer Y-axis readings (left) indicates that standing behaviour is characterized by a larger mean (denoted by a grey triangle) than those recorded with the animal lying down. Accelerometer Z-axis readings (right), indicates that standing position have a larger interquartile range than the lying ones.

To achieve “a low-memory algorithm”, the accelerometer data is resampled from 17.6 Hz to 0.27 Hz as our experiments have proven that this data rate is enough for the algorithm. This way, memory requirements during the sliding window operations decreases and battery life of the device increases. Based on these appreciations and given the series of readings from the Y-axis of the accelerometer at discrete timestamps $t_{i}$, denoted by $f_{ay}\left(t_{i}\right)$*,* the following algorithm has been developed:

1. First, a rectangular sliding window of the last 15 min is used to count the number of readings in the Y-axis between a given superior and inferior threshold, $thr_{sup}\;$and $thr_{inf}$, respectively. The values of these thresholds are obtained from the distribution shown in Figure 7 to maximize the difference in the resulting count while standing and lying. This operation results in a function $f_{count}\left(t\right)$ (1) given by:
  (1) $$f_{count}\left(t\right)=\overset{t}{\underset{i=t-15\;minutes}{\sum }}\left[thr_{inf}<f_{ay}\left(i\right)<thr_{sup}\right],$$
2. Next, $f_{count}\left(t\right)$ is thresholded to obtain a binary signal $f_{standing}\left(t\right)$ (2) depending on the value of the function in each instant relative to a threshold $thr_{standing}$. This way, any value greater than $thr_{standing}\;$will be denoted as 1 (standing), while values smaller than the threshold will be converted to 0 (lying).
  (2) $$f_{standing}\left(t\right)=\{\begin{array}{c} 1\;\left(standing\right)\;\;\;\;\;if\;f_{count}\left(t\right)>thr_{standing}\;\\ 0\;\left(lying\right)\;\;\;\;\;\;\;\;\;\;\;\;if\;f_{count}\left(t\right)\leq thr_{standing} \end{array},$$
3. This binary function $f_{standing}\left(t\right)$ is converted to a discrete transition signal $f_{lb}\left(t\right)$ (3) taking the absolute value of the difference between $f_{standing}\left(t\right)$ at any given time and its immediately previous value with each transition from standing to lying down and vice-versa represented by a 1.
  (3) $$f_{lb}\left(t\right)=\left|f_{standing}\left(t\right)-f_{standing}\left(t-1\right)\right|,$$
4. Finally, with this discrete transition signal $f_{lb}\left(t\right)$ computed, another rectangular sliding window is used to count the number of transitions that took place in the previous 5 h of each reading. This function $f_{parturition}\left(t\right)$, acts as a proxy to predict parturition based on the lying bouts occurrence increasement before calving.
  (4) $$f_{parturition}\left(t\right)=\sum_{i=t-5\;hours}^{t}f_{lb}\left(i\right),$$

## 3. Results and Discussion

**Figure 8:**
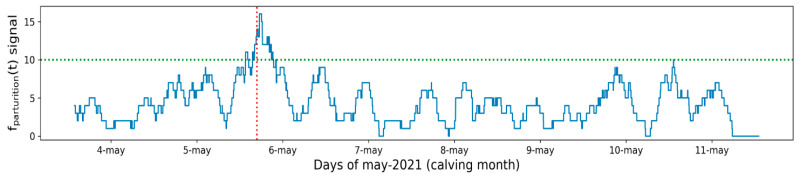
Number of lying bouts (function $f_{parturition}\left(t\right)$), in the last five hours calculated with a rolling window for cow 03 for a week. Dotted red line signalizes the corresponding calving instant. Dotted green line denotes a candidate value to detect the parturition event.

As indicated in Table 2, cow number 03 calved on 5 May 2021 at 4:45 PM. Figure 8 shows the values of function $f_{parturition}\left(t\right)$ in the last five hours calculated with a rolling window for cow 03, for a week (from 4 May to 11 May), using the proposed algorithm for parturition prediction. A notable increase of this function is observed near the parturition instant, signalled with a vertical red dotted line.

Figure 8 represents the function $f_{parturition}\left(t\right)$ during a week. This figure shows that it is during the hours before calving (2~3 h) when this function takes the highest values, reaching the maximum at the instant of calving. Furthermore, a horizontal dotted green line in Figure 8 denotes a value that is only surpassed during the calving event ($f_{parturition}\left(t\right)$ = 10). This value could be used as a trigger of the parturition detection for this cow 03. It is noted that this signalled value differs between individuals since there is variance between the activity and energy expenditure of each animal, and therefore this value has to be dynamically calculated (for each cow) on the collar based on previous readings.

**Figure 9:**
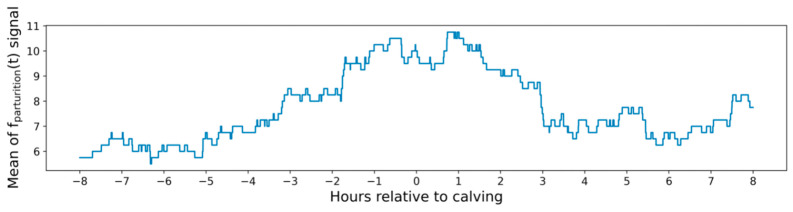
Mean of lying bouts (function $f_{parturition}\left(t\right)$) from the five cows which parturitions are indicated in Table 2.

As indicated in Table 2, during the data collected over a period of eleven months (August 2020–July 2021), five cows calved. Figure 9 shows the mean of function $f_{parturition}\left(t\right)$ in the last five hours calculated with a rolling window, and generated from the algorithm showed before (from the five calving events that took place). To calculate this mean, the values of this function have been aligned on the moment of parturition (0 h relative to calving). It is observed in Figure 9 that the mean value of the function $f_{parturition}\left(t\right)$ increases two hours before the calving of the cows. This increase allows us to determine that the cow is close to parturition. As previously mentioned, the parturition trigger value of 10 signalled in Figure 8 is only applicable to cow 03. This can be shown in Figure 9, where the $f_{parturition}\left(t\right)$ signals from the rest of the cows have brought the signal mean value slightly down.

The heuristic-based algorithm we have developed traces lying bouts with enough resolution to detect their increase in a time frame, which can be used to detect cow calving. This is accomplished without the need to calculate a calving indicator index that requires tracking the step count and the time spent lying down by the animal as in [27], mainly due to this variables stability when compared with the number of lying bouts near calving.

A very important aspect of the developed calving detection algorithm is its simplicity. This algorithm must be programmed in the microcontroller housed in the collar placed on the cow’s neck, which represents a significant limitation in the microcontroller’s computing and memory fields. Furthermore, a coarse estimate of the calving date in the receiving end of the calving prediction is also useful to use in conjunction with the algorithm, as it ensures the rejection of any naturally inviable false positive from the algorithm (i.e., calving detection one month before and after mount).

The dataset used in previous studies [27,40], usually includes data in a small temporal window around calving (1–5 days). The data collected in our study enable the back test of algorithms in a much more extensive temporal window, something essential to validate any algorithm that would run in a real-world environment, such as the proposed sensorized wearable device.

Although the proposed algorithms of this study are focused on calving prediction, the developed sensorized wearable device and the collected data enable the development of different algorithms that could be of great help to farmers both in extensive and intensive livestock. Additionally, the generalist design of the presented wearable device could be equally helpful to develop hardware solutions oriented to different animals, benefiting their caretakers with the localization data from the collar, the suite of sensors that it incorporates, and other algorithms that could be implemented within the device.

## 4. Conclusions and Future Work

In this paper, a low-cost neck-mounted sensorized wearable device to continuous long-term monitoring cow data has been presented. To incorporate the ability to detect cow parturitions with enough anticipation using only this device an algorithm has been developed. This algorithm can detect an increment in the number of times the cow stands up and lies down (lying bouts) using a signal calculated from the accelerometer’s readings. To test the proposed algorithm, data collection using the cow neck-mounted devices and annotations from in-situ human observers has been carried out for eleven months (August 2020–July 2021). The data gathered by the neck-mounted collars correspond to six different cows monitoring in extensive livestock farming.

This preliminary study (of six cows, with five calving events), provides evidence that cow approaching parturition shows an increase in lying bouts behavioural pattern that can predict calving on average two hours before calving. To confirm these results, however, more pregnant cows need to be monitored and further research is required to refine this algorithm. The long-term character of the data acquired will allow for individualization of the thresholds for calving detection by calculating the baseline “restlessness level” of a particular animal. This could be used by the collar to generate an alert system to warn the farmer of the onset of calving.

The low-cost of this device (≈100 € for small-scale production) would benefit most large livestock holdings by greatly reducing the number of hours the human experts must manually monitor individual animals. Economies of scale would allow the unitary price of the collar to be lowered even more, which would allow massive adoption even for the monitoring of large herds. However, smaller farms with less resources would also benefit from the reduced cost of the device, which lowers the entry barrier into a technology such as this, thus allowing for its adoption.

Future work will focus in two separate points. The first one, data related, requires the acquisition of calving data from a larger number of cows to validate the developed algorithm and to develop a new data driven algorithm that learns from the available data from different cows (both from the same breed and from different breeds, to deal with the variance between different species). This new algorithm could be dedicated to the classification of standing and lying behaviour or to the detection of the birth event as a time series task. Furthermore, the data already gathered from the animals long before calving and therefore related mostly to the normal activity of the animal would allow for the development of algorithms that analyse cow behaviour (grazing, ruminating, etc). A deviation of the normal behaviour of a particular animal due to sickness, heat, or an abortion could be signalised to the farmer.

The second point englobes the development of a new neck-mounted collar with IMU, GNSS and wireless low energy, and long-range communication using the LoRa protocol. This wireless communication would allow farmers to physically localize cows in extensive livestock farming, as well as receiving notifications of the detected parturition event, reducing the workload associates with parturition, and preventing dystocia in unattended calving. The energy autonomy of the device is an aspect of great importance in terms of its practical utility. In the tests carried out, it has been identified as an improvement parameter. For this, solar-powered technology is being incorporated in order to increase overall autonomy.

## Figures and Tables

**Table 1:** Raw data collected by the sensorized collar and saved in the microSD card.

Variable	Datatype	Units
Timestamp	int32	ms since Unix Epoch
Temperature IMU	float32	degrees C
Temperature DB18B20	float32	degrees C
Longitude (Lon)	int32	degrees (×10^−7^)
Latitude (Lat)	int32	degrees (×10^−7^)
Altitude above sea level (Alt)	int32	m (×10^−3^)
Speed	int32	m s^−1^ (×10^−3^)
Acceleration axis X (ax)	float32	×g
Acceleration axis Y (ay)	float32	×g
Acceleration axis Z (az)	float32	×g
Rotation X axis (gx)	float32	degrees s^−1^
Rotation Y axis (gy)	float32	degrees s^−1^
Rotation Z axis (gz)	float32	degrees s^−1^

**Table 2:** Cow-wise distribution of the data.

Cow	Raw Data Collection Period(dd/mm/yyyy)	Calving Date and Hour	Hours of Raw Data	Hours of Labelled Data
01	24/08/2020–17/02/2021	01/12/2020 13 h:30′	1.634	212
02	24/08/2020–25/05/2021	24/02/2021 08 h:30′	3.417	510
03	01/03/2021–15/06/2021	05/05/2021 16 h:45′	1.720	279
04	05/10/2020–12/07/2021	25/05/2021 13 h:35′	2.957	470
05	08/02/2021–30/07/2021	11/07/2021 20 h:15′	1.130	159
06	24/08/2020–27/01/2021	-	1.887	147
Total	24/08/2020–30/07/2021	-	12.745	1.777

**Table 3:** Schema of raw data collected by the collars.

	DB18B20	GNSS				IMU						
Timestamp	Temp	Lon	Lat	Alt	Speed	ax	ay	az	gx	gy	gz	Temp

**Table 4:** General behaviour annotations.

ID	Action
A1	Grazing-Eating
A2	Ruminating
A3	Neutral
A4	Walking

**Table 5:** Standing/lying behaviour annotations.

ID	Action
B1	Standing
B2	Lying

## Data Availability

Not applicable.
